# Improving the Effect of Cancer Cells Irradiation with X-rays and High-Energy Protons Using Bimetallic Palladium-Platinum Nanoparticles with Various Nanostructures

**DOI:** 10.3390/cancers14235899

**Published:** 2022-11-29

**Authors:** Bartosz Klebowski, Malgorzata Stec, Joanna Depciuch, Agnieszka Panek, Dawid Krzempek, Wiktor Komenda, Adrianna Gałuszka-Bulaga, Anna Pajor-Swierzy, Jarek Baran, Magdalena Parlinska-Wojtan

**Affiliations:** 1 Institute of Nuclear Physics Polish Academy of Sciences, 31-342 Krakow, Poland; 2 Department of Clinical Immunology, Jagiellonian University Medical College, 30-663 Krakow, Poland; 3 Jerzy Haber Institute of Catalysis and Surface Chemistry Polish Academy of Sciences, 30-239 Krakow, Poland

**Keywords:** palladium nanoparticles, platinum nanoparticles, bimetallic nanoparticles, green chemistry, gallic acid, radiosensitizers, cancer treatment, proton irradiation, X-ray irradiation, MTS test

## Abstract

**Simple Summary:**

Radiation-based anticancer therapies are often ineffective as cancer cells may acquire radioresistance. Moreover, such therapies can cause unwanted side effects, affecting normal tissues. This problem may be remedied by the use of nanometric radiosensitizers. These compounds in non-toxic concentrations improve the effectiveness of therapy while reducing the total dose of radiation used. In this work, we checked the radiosensitizing properties of bimetallic palladium-platinum nanoparticles with different nanostructures: nano-alloy and core-shell. It has been found that nano-alloy structures are more promising radiosensitizers in vitro, and their effect is more satisfactory when using X-rays than high-energy protons. Thus, by appropriately designing the microstructure of nanomaterials, it is possible to modulate their radiosensitizing potential and enhance the therapy′s effectiveness.

**Abstract:**

Nano-sized radiosensitizers can be used to increase the effectiveness of radiation-based anticancer therapies. In this study, bimetallic, ~30 nm palladium-platinum nanoparticles (PdPt NPs) with different nanostructures (random nano-alloy NPs and ordered core-shell NPs) were prepared. Scanning transmission electron microscopy (STEM), selected area electron diffraction (SAED), energy-dispersive X-ray spectroscopy (EDS), zeta potential measurements, and nanoparticle tracking analysis (NTA) were used to provide the physicochemical characteristics of PdPt NPs. Then, PdPt NPs were added to the cultures of colon cancer cells and normal colon epithelium cells in individually established non-toxic concentrations and irradiated with the non-harmful dose of X-rays/protons. Cell viability before and after PdPt NPs-(non) assisted X-ray/proton irradiation was evaluated by MTS (3-(4,5-dimethylthiazol-2-yl)-5-(3-carboxymethoxyphenyl)-2-(4-sulfophenyl)-2H-tetrazolium) assay. Flow cytometry was used to assess cell apoptosis. The results showed that PdPt NPs significantly enhanced the effect of irradiation on cancer cells. It was noticed that nano-alloy PdPt NPs possess better radiosensitizing properties compared to PtPd core-shell NPs, and the combined effect against cancer cells was c.a. 10% stronger for X-ray than for proton irradiation. Thus, the radio-enhancing features of differently structured PdPt NPs indicate their potential application for the improvement of the effectiveness of radiation-based anticancer therapies.

## 1. Introduction

More effective anticancer therapies are of priority for current medicine due to the increasing incidence of cancer worldwide [1]. Currently available methods of cancer treatment are often related to the harmful effects on normal cells and acquisition of tumor resistance to chemotherapeutics or radiotherapy, thus forcing the search for innovative, alternative methods of eliminating neoplasms [2,3]. On the other hand, immunotherapy, characterized by high selectivity of action, is still not a routine form of cancer treatment [4]. 

Radiation-based anticancer therapies, such as X-ray-, gamma ray-, proton- or boron neutron capture therapy, are used for the local treatment of tumors [5,6,7]. The use of individual radiotherapy mode depends, among others, on the location of cancer. Radiation therapies are used both in neoadjuvant treatment—to shrink the tumor before surgery, in adjuvant, or in palliative analgesic treatment [8,9]. Photon, proton or neutron radiation can interact with the cell DNA directly or indirectly. About 80% of radiation-based damage is caused by the direct effect. This effect occurs when radiation interacts with water (the main component of cells), leading to the formation of reactive oxygen species (ROS) destructive to DNA. In turn, the indirect effect is based on the interactions with atoms or molecules of cells (DNA, proteins, and even lipids), without an intermediate step being the interaction with water [10,11]. 

**Figure 1:**
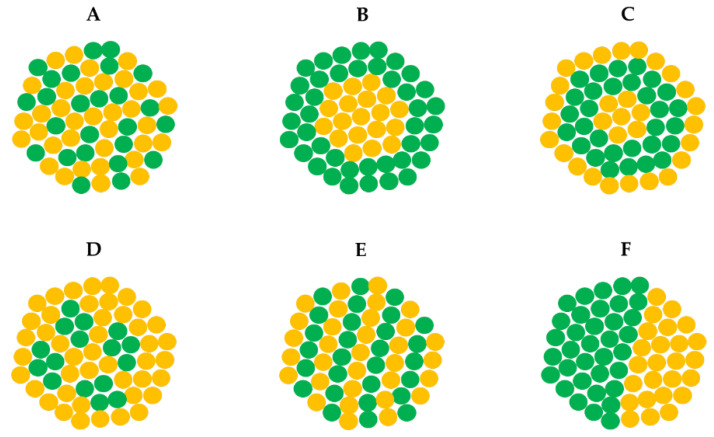
Different types of bimetallic nanoparticles: (**A**) alloyed, (**B**) core-shell, (**C**) multishell core-shell, (**D**) multiple core materials coated by a single shell, (**E**) intermetallic and (**F**) subclusters. The two types of metal atoms are marked in green and yellow. Modified from [25].

The effectiveness of radiotherapy can be enhanced by the use of nano-sized radiosensitizers. For this purpose, particularly noble metal nanoparticles (NPs) are suitable due to their stability, ease of functionalization, high ratio of surface area to volume, as well as the possibility of their synthesis without the use of organic reagents [12]. Importantly, such nano-radiosensitizers should be neutral towards the biological systems (e.g., cells) at low concentrations. The high-Z metal NPs sensitize the cells to subsequent radiation-based anticancer treatment, and a joined anticancer effect can be observed. Various mechanisms (physical, chemical-biological, or biological) are involved in radiosensitization; these mechanisms can overlap or act individually, depending, for example, on the type or energy of radiation [13,14]. The final effect of radiosensitization by NPs depends on the type of metal used, its size, shape, and the method of synthesis [15,16,17,18]. Exemplary, the generation of ROS by cells under the influence of NPs has already been known in the literature, e.g., for the widely used gold nanoparticles [19]. There are also studies on the use of multicomponent metallic NPs for the improvement of radiotherapy effectiveness, such as gold-silver [20], gold-platinum, gold–palladium [21], gold-zinc [22] or gold-silver nanocomplexes [23]. The combined effect of metals was seen not only with respect to human cells but also in bacteria treated with bimetallic silver-zinc, silver-cobalt, silver-nickel or silver-copper NPs [24]. However, it has not yet been verified whether the nanostructure of bimetallic NPs may influence their radiosensitizing properties. Exemplified different types of bimetallic NPs are shown in Figure 1. 

Thus, in this work, the effect of the nanostructure of bimetallic palladium-platinum (PdPt NPs) on their radiosensitizing properties in vitro was evaluated. For our research, two forms of radiation were selected– X-rays and high-energy protons, and two types of bimetallic PdPt NPs were synthesized. The first type of PdPt NPs were the nano-alloy ones (PdPt I), characterized by irregular, randomly distributed platinum and palladium atoms within the NPs. The second type was the core-shell structured NPs (PdPt II), in which palladium is in the core, surrounded by the platinum layer. In this case, a clear segregation of individual atoms was visible. The selection of such bimetallic structures was not accidental. In order to reliably compare the radiosensitizing properties of the obtained nanoparticles, they should not only have a similar shape, size and surface charge, but also the method of their synthesis (especially the used reducing agents and stabilizers) ought to be almost the same method. Using wet chemistry, we were able to easily synthesize two types of bimetallic NPs: nano-alloy and core-shell NPs. Obtaining other bimetallic structures (presented in Figure 1) by the same method with the same reagents would be impossible. The selected two types of bimetallic NPs will ensure that biological systems (such as cells) come into direct contact with both platinum and palladium (for PdPt I) and only platinum (for PdPt II), which will allow us to assess whether there will be combined effect of both metals on cells for PdPt I. The NPs were obtained by the green chemistry method, using gallic acid as both the reducing and the stabilizing agent. Then, the morphology (size and shape), structure and chemical composition of PdPt NPs were evaluated by scanning transmission electron microscopy (STEM), selected area electron diffraction (SAED) and energy-dispersive X-ray spectroscopy (EDS). Additionally, the zeta potential values of NPs in a wide pH range were determined, and nanoparticle tracking analysis (NTA) was used to obtain information about the size distribution and the concentration of PdPt NPs. In parallel, biological tests were performed using two colon cancer cell lines with different aggressiveness (SW480 and SW620 cells), as well as a normal colon epithelial cell line used as a control (CCD 841 CoN cells). The MTS (3-(4,5,-dimethylthiazol-2-yl)-5-(3-carboxymethoxyphenyl)-2-(4-sulfophenyl)-2H-tetrazolium) test was used to evaluate the cell viability after the treatment with different types of PdPt NPs, X-ray- or proton irradiation, as well as after combined PdPt NPs-assisted irradiations. 

## 2. Materials and Methods

### 2.1. Reagents and Chemicals

Hydrogen hexachloroplatinate (IV) hexahydrate (H_2_PtCl_6_ ∙ 6H_2_O), palladium (II) chloride (PdCl_2_) and gallic acid (C_6_H_2_(OH)_3_CO_2_H) were obtained from Sigma-Aldrich (Saint Louis, MO, USA). All reagents were used without any additional purification or modification steps.

### 2.2. Synthesis of Bimetallic PdPt NPs

#### 2.2.1. PdPt Nano-Alloy Synthesis (PdPt I NPs)

PdPt nano-alloy was obtained by green chemistry method using gallic acid as a stabilizer and reducing agent. Before the reaction, it was necessary to convert the palladium (II) chloride to hydrogen tetrachloropalladate (H_2_PdCl_4_) by an equivalent of hydrochloric acid (HCl). For the synthesis of PdPt (I) NPs, 0.25 mL 10 mM aqueous solutions of H_2_PtCl_6_ and H_2_PdCl_4_ and 17.5 mL of distilled water were mixed in a round-bottom flask. The reaction mixture was heated until boiling on a magnetic stirrer (400 rpm), and then 2 mL of 5 mM freshly prepared aqueous solution of gallic acid was poured into the mixture. The reaction was carried out for 45 min. The raw NPs solution was purified by centrifugation in fresh water three times (20,000× *g*, 10 min). 

#### 2.2.2. PdPt Core-Shell Synthesis (PdPt II NPs)

The synthesis of PdPt core-shell NPs was also carried out using gallic acid. To prepare a core-shell structure, 17.5 mL of distilled water and 0.25 mL aqueous solution of H_2_PdCl_4_ were mixed in a round-bottom flask and heated to the boiling point. Then, a fresh portion of the aqueous gallic acid solution (2 mL, 5 mM) was added, and the reaction was continued for 45 min. Then, 0.25 mL aqueous solution of H_2_PtCl_6_ was added, and the reaction was carried out for another 45 min. The obtained NPs were purified by centrifugation, as described in Section 2.2.1. 

### 2.3. TEM Characterization

Scanning transmission electron microscope (STEM) with a high-angle annular dark-field detector (HAADF) operating in conventional mode was used to evaluate the morphology of the synthesized PdPt NPs. Selected area electron diffraction (SAED) patterns were taken in the TEM mode to determine the local crystallinity of the NPs. All the measurements were performed on an aberration-corrected FEI Titan electron microscope (Hillsboro, OR, USA) operating at 300 kV equipped with a FEG (field emission gun) cathode. Energy-dispersive X-ray spectroscopy (EDS) was used to identify the local chemical composition of bimetallic PdPt NPs. The EDS mappings were done by an FEI Talos TEM (Waltham, MA, USA) operating at 200 kV equipped with a FEG cathode and four in-column EDS detectors (Super EDS system). The PdPt NPs size distribution was evaluated based on the STEM images and EDS maps taken from different areas of the TEM grids, showing that the morphology of NPs is the same within the sample. Because the shape of PdPt NPs was not perfectly spherical, the diameter of PdPt (I) NPs was measured as a distance between the two most distant points of these NPs. For PdPt (II) with phase segregation, it was possible to determine the diameter of the palladium core and thickness of the platinum shell. All TEM analyzes were performed twice to check whether the applied methodology of NPs synthesis was repeatable.

### 2.4. Zeta Potential Measurements

The zeta potential distribution, in the pH range from 3 to 11, of both types PdPt NPs was determined by the microelectrophoretic method using Zetasizer Nano Series from Malvern Instruments (Worcestershire, UK). The Smoluchowski model was used in zeta potential measurements. Each value was obtained as an average of three subsequent runs of the instrument with at least 20 measurements. All experiments were performed in water at room temperature. 

### 2.5. Nanoparticle Tracking Analysis (NTA)

Nanoparticle tracking analysis was conducted using NanoSight LM10-HS488FT14 Nanoparticle Characterization System from Malvern (Worcestershire, UK). Briefly, 1 µL of PdPt NPs solution was diluted 1000× to a total volume of 1 mL in distilled water. Then, 600 µL of the NPs solution was loaded into the measuring chamber using an insulin-type syringe. The syringe was mounted onto the pump, and the sample flowed at a constant flow rate of 80 units. Three one-minute videos were recorded, and samples were analyzed using NanoSight NTA 3.0 analytical software (Malvern Instruments, Worcestershire, UK). By this method, the diameter of PdPt NPs, as well as their concentrations (NPs/mL), were estimated. The measurement was performed in triplicate for each sample.

### 2.6. Irradiation Simulation Protocols 

#### 2.6.1. X-ray Irradiation

For X-ray irradiation, the Philips X-ray machine model MCN 323 (Hamburg, Germany), working at 250 kV and 10 mA, was used. The total dose delivered to the cell culture amounted to 15 Gy. The cells in multi-well plates were irradiated at room temperature on a special phantom. The dimensions of the radiation field were 20 × 20 cm, and the source to the surface distance was 34.8 cm. The dosimetry was made using the earlier referenced dosimeter (PTW UNIDOS and PTW TM31013 ionization chamber). Dosimetry measurements for these experimental conditions indicated a dose rate of 0.03 Gy/s. 

#### 2.6.2. Proton Irradiation

Proton irradiations were performed using the IBA Proteus C-235 isochronous cyclotron (Louvain-la-Neuve, Belgium) with a compact conventional magnet and two gantries equipped with a scanning nozzle. For these studies, pencil beam scanning techniques were applied. Irradiations were performed at room temperature using a monoenergetic field with an energy of 225 MeV and dimensions of 20 cm × 20 cm. The cells were irradiated at 1.1 cm water equivalent depth with a dose of 15 Gy (both for protons and X-rays). This dose was selected because it was non-toxic for the used cell lines; previously, experiments to determine the dose-response effect for individual cell lines were performed. Proton irradiation was preceded by dosimetry measurements performed with Markus’s type ionization chamber calibrated with respect to the dose absorbed by the water. After the proton or X-ray irradiation, the cells of each line were incubated for 18 h, and then a viability test was performed.

### 2.7. Cell Culture

Two colon cancer cell lines (SW480 and SW620), as well as the fetal colon cell line CCD841 CoN (CRL-1790), were obtained from the American Type Culture Collection (ATCC, Manassas, VA, USA). SW480 and SW620 cells were cultured in DMEM (Dulbecco’s modified eagle medium) with high glucose (Corning, Corning, NY, USA). CCD841 CoN cells were cultured in EMEM (Eagle’s minimum essential medium). All media were supplemented with 10% fetal bovine serum (FBS) and ciprofloxacin (10 µg/mL). The cells were cultured by bi-weekly passages in a 37 °C humidified atmosphere with 5% CO_2_ and regularly tested for *Mycoplasma* sp. contamination by PCR-ELISA kit (Roche, Mannheim, Germany) according to the manufacturer’s instructions. 

### 2.8. MTS Assay

The MTS (3-(4,5,-dimethylthiazol-2-yl)-5-(3-carboxymethoxyphenyl)-2-(4-sulfophe- nyl)-2H-tetrazolium) test (CellTiter 96^®^ Aqueous One Solution Cell Proliferation Assay, Promega, Madison, WI, USA) was used for the assessment of cell viability cultured with PdPt NPs and/or irradiated with X-ray or protons. Briefly, the cells were cultured in flat-bottom 96-well plates (Sarstedt, Numbrecht, Germany) at a density of 10^4^ cells per well in a medium containing 10% FBS. After 48 h of culture, 20 µL of PdPt NPs were added to 100 µL medium with cells, giving the final NPs concentration in medium from 5 to 150 µg/mL. After an incubation period of 18 h, 20 µL of MTS dye solution was added per well. The quantity of formazan product was detected by absorbance measurements with a 96-well plate reader (Spark^®^ Tecan, Mannedorf, Switzerland). The cell viability can be calculated by measuring the ratio of the absorbance of treated cells to the absorbance of the control. Each sample was measured three times. The absorbance was measured from 1.5 to 3 h (depending on the used cell line) after the addition of the MTS. The measurements were stopped when the absorbance of the control exceeded the value of 2.0. For further PdPt NPs-supported irradiation research, their maximum concentration, which does not cause a significant decrease in cell viability (not higher than 15%), was selected (50 µg/mL and 75 µg/mL for SW480 and SW620 cells, respectively). In turn, normal CCD 841 CoN (control) during X-ray/proton irradiation were cultured with both (50 and 75 µg/mL) concentrations allowing a reliable comparison of the effect of the PdPt NPs-assisted irradiation on normal and colon cancer cells. 

### 2.9. Annexin V-Binding Assay

Analysis of cells apoptosis was performed by Annexin V-binding assay (Fluorescein isothiocyanate (FITC) Annexin V apoptosis detection Kit I (BD, Pharmingen, San Diego, CA, USA)), as described previously [20]. Cells were plated in a 24-well plate at a density of 10^6^/well. After 48 h of incubation, the normal and cancer cells were exposed to PdPt NPs and irradiated with X-rays/protons. The medium from each well was transferred in a pre-labeled separate centrifuge tube. Adherent cells from the same well were then trypsinized and transferred to the same tubes. All samples were centrifuged, and the supernatant was discarded. Pellets were re-suspended in a solution containing Annexin V-FITC conjugated and incubated for 10 min in the dark prior to flow cytometry analysis. Annexin V-binding assay was performed on a FACS Calibur flow cytometer (BD Biosciences, Immunocytometry Systems, San Jose, CA, USA). The results were analyzed using a FACS Diva v.8.1. software (BD Biosciences, San Jose, CA, USA).

### 2.10. Statistical Analysis

The obtained zeta potential values and MTS assay results are shown as the means + SEM (standard error of the mean). The data were analyzed by one-way analysis of variance (ANOVA) followed by the post hoc Tukey test. Statistical significance was indicated when the *p*-value < 0.05. The data were presented graphically using GraphPad Prism 8 Software. 

## 3. Results and Discussion

### 3.1. Mechanism of PdPt NPs Synthesis

**Figure 2:**
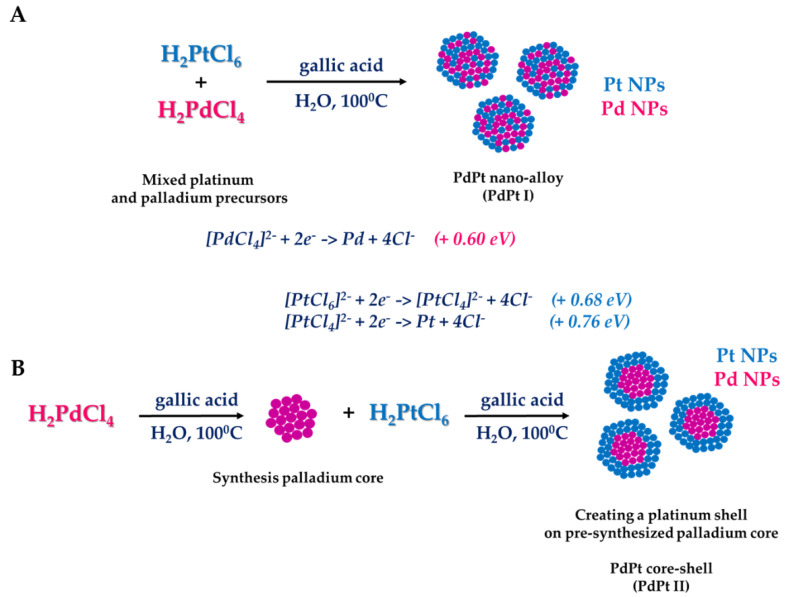
Green synthesis mechanism of: (**A**) PdPt nano-alloy and (**B**) PdPt core-shell NPs, considering the electrochemical potentials of individual reactions.

Both types of bimetallic PdPt NPs were obtained by green chemistry method using cheap and eco-friendly gallic acid. Gallic acid (3,4,5-trihydroxybenzoic acid), due to its chemical structure, can be a universal reducing agent. The structure of gallic acid includes an aromatic ring with three hydroxyl groups and one carboxyl group as a substituent. As a result, gallic acid tends to form chelating rings with various metal ions derived from metal precursors. During the NPs synthesis process, gallic acid itself is oxidized to benzoquinones [26,27,28]. Importantly, gallic acid also acts as a stabilizer of these reactions; it prevents the assembly of NPs into larger agglomerates. The proposed mechanism for obtaining both types of bimetallic PdPt NPs is presented in Figure 2.

The synthesis of nano-alloy bimetallic PdPt NPs (PdPt I) is a one-step process (Figure 2A). The formation of the nano-alloy structure can be explained by the values of the electrochemical potentials of individual metals. As can be seen in the diagram below (Figure 2), the standard electrochemical potentials for the platinum and palladium precursors are very similar. Thus, the simultaneous introduction of these metal precursors into the reaction mixture, followed by the addition of the reducing agents, will result in an almost simultaneous reduction of both metals. This reaction takes place quickly–immediately after adding gallic acid, we observed a change in the color of the solution, suggesting the formation of bimetallic NPs. However, the reaction was continued for a longer time to ensure that all of the precursors were reacted. Therefore, we did not observe the differentiation of phases in these NPs; palladium and platinum were randomly distributed within the NPs.

To obtain a core-shell PdPt structure (PdPt II), a different strategy was used (Figure 2B). At first, we reduced the palladium precursor with an aqueous solution of gallic acid to obtain the palladium cores. This reaction took a relatively long time because–compared to the synthesis of the nano-alloy structures–we do not have here the driving force in the form of the differences in electrochemical potentials of two metals, which significantly accelerate the reaction process, as already demonstrated [29]. In the second step, we added the platinum precursor to the previously formed Pd NPs. An immediate color change of the solution and the formation of bimetallic core-shell nanostructures were observed. 

### 3.2. Morphology, Structure, Chemical Composition, Zeta Potential and NTA of PdPt NPs

**Figure 3:**
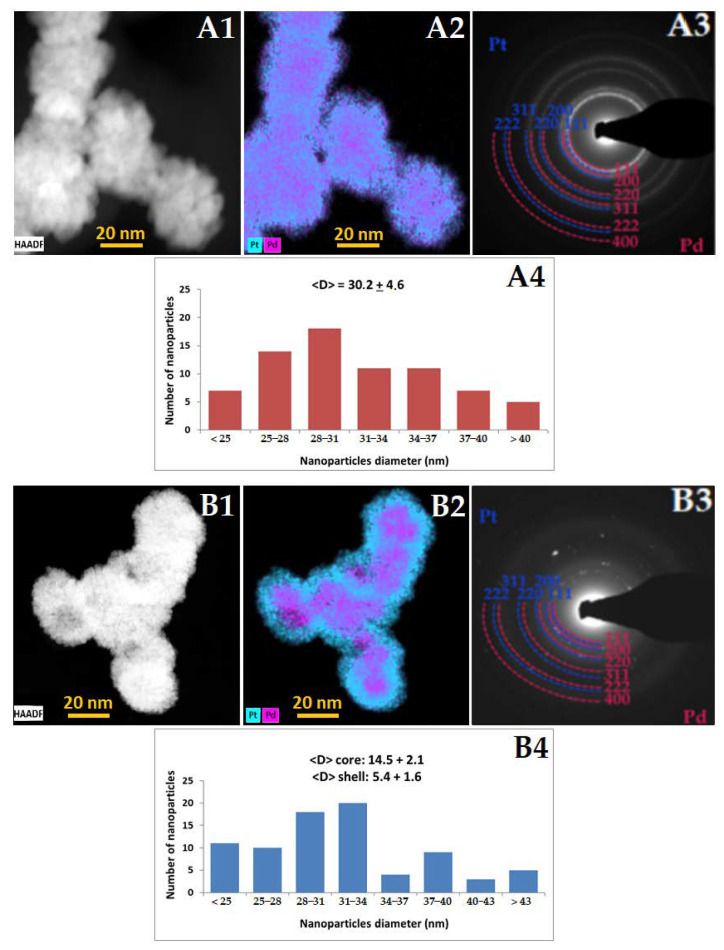
STEM HAADF images of (**A1**) PdPt (I) and (**B1**) PdPt (II) NPs with the corresponding (**A2**,**B2**) EDS distribution maps, (**A3**,**B3**) SAED patterns indexed with lattice parameters of palladium and platinum and (**A4**,**B4**) size distributions (diameter of NPs/core/shell ± standard error).

The morphology (size and shape), structure, as well as chemical composition of PdPt NPs were evaluated by electron microscopy. STEM images, SAED patterns with interpretation and EDS maps for PdPt (I) and PdPt (II) NPs are depicted in Figure 3. On the basis of STEM photos (Figure 3A1,B1), it can be seen that the obtained PdPt NPs have (approximately) a spherical shape, and their size can be estimated to be about 30 nm in both cases (Figure 3A4,B4). Additionally, for PdPt (II) NPs, where there is a clear boundary between the palladium core and the platinum shell (Figure 3B2), it is possible to determine the approximate diameter of the core (~15 nm) and the thickness of the shell (~7.5 nm). It should be mentioned that not all NPs were perfectly spherical, and therefore, conventionally, the diameter was defined as the distance between the two most distant points of the NPs. PdPt (I) NPs form nanoslave-like structures; they consist of ultra-small (~2–3 nm) Pd or Pt NPs agglomerating into larger structures. EDS maps (Figure 3A2,B2) confirmed that we obtained both: nano-alloy structures with irregular distribution of individual platinum and palladium, as well as a core-shell structure, where palladium is the core, surrounded by a platinum layer. From the SAED patterns (Figure 3A3,B3), it was concluded that PdPt (I) and PdPt (II) NPs are characterized by crystalline nanostructure because in both cases, there are clear, non-blurred diffraction rings. The diffraction rings can be attributed to the (111), (200), (220), (331), (222) and (400) lattice planes of Pt and Pd nanocrystals crystallized in the face-centered cubic structure [30,31]. The rings from Pt and Pd almost overlap due to the slight differences in the lattice parameters (a_Pd_ = 0.389 nm and a_Pt_ = 0.392 nm). 

The results of the measurements of the zeta potential of PdPt (I) and PdPt (II) NPs as a function of pH are summarized below in Table 1.

Analysis of the zeta potential provides important information on the stability of the NPs suspension. NPs with a high negative or positive value of this potential showed a higher tendency to repel each other and therefore did not agglomerate. NPs with a zeta potential in the range from −10 mV to 10 mV have generally reduced stability; low zeta potential values may (in the short or long term) result in agglomeration or flocculation of NPs, due to the attractive Van der Waals forces acting on them [32]. Negative values of the zeta potential for these NPs were expected, as gallic acid (stabilizer of NPs) and its oxidation products are negatively charged [21,26]. For the tested pH range, the zeta potential of PdPt (I) decreased up to pH = 7 (reaching a minimum of c.a. −16 mV) and then increased again to stabilize at the level of about −11 mV. On the other hand, for PdPt (II) NPs, the zeta potential value decreased with increasing pH very slowly. Thus, the solution of PdPt (I) NPs tends to be more stable than PdPt (II). It is worth noting that the solution of both types of NPs showed stability even after six months after their synthesis (the TEM photos did not show the formation of NPs agglomerates). Presumably, these nanosystems will show the least pH-dependent stability at acidic pH. This is important because, generally, cancer cells have a slightly lower pH (6.4–7.0) than normal cells (7.2–7.5) [33]. Therefore, in order to ensure the highest possible stability of such NPs, functionalization with compounds that significantly changed the value of the zeta potential so that it would be above +20 mV or below –20 mV would be considered. What’s more, the zeta potential value plays a key role in the initial absorption of NPs onto the cell membrane [34]. 

**Figure 4:**
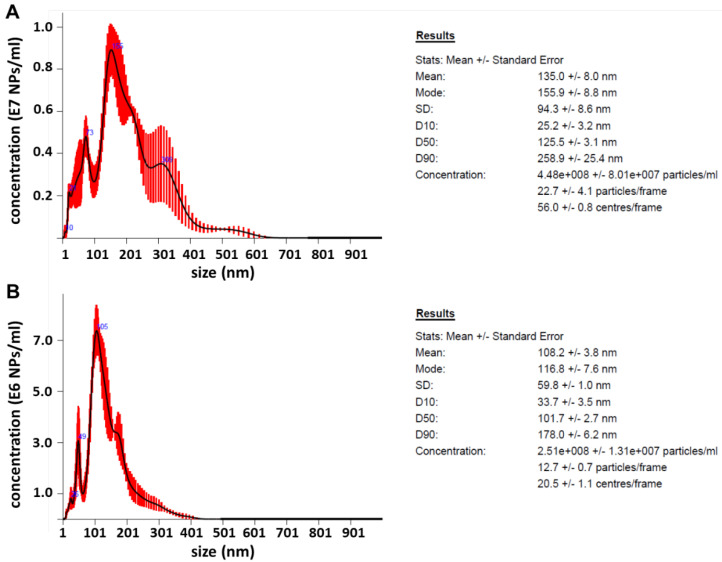
Nanoparticle tracking analysis results for (**A**) PdPt (I) and (**B**) PdPt (II) NPs.

The NTA allowed the assessment of the size distribution (determined from the Stokes--Einstein equation based on the difference in Brownian motions of NPs with different sizes) and count of the PdPt NPs number in solution. The NTA results of the PdPt NPs diluted in water are shown in Figure 4. 

However, the estimated diameter of PdPt (I) and PdPt (II) NPs significantly differed from those determined directly by STEM. In both cases, these results were overestimated by several times. The reason for such results may be that both types of NPs (Figure 3A1,B1) tend to form clusters very close to each other. Presumably, the NTA software counts several NPs next to each other as one large cluster; hence such inflated diameter results were obtained. Moreover, the NTA results were not improved by the earlier sonication of the samples, the change of the solution in which the NPs were suspended, or the regulation of NPs flow rate. It should also be noted that the apparatus used in this experiment has a resolution of 10 nm. The obtained PdPt NPs are indeed larger but still at the edge of the apparatus resolution. Therefore, the NPs concentrations determined by this method were unreliable. For the PdPt NPs synthesized, the actual concentrations (µg/mL) were determined by heating a known volume of purified NPs solution in a laboratory oven and then weighing the dried NPs. 

### 3.3. Changes in Viability of Colon Cancer and Normal Cells Induced by the Treatment with PdPt NPs Only, or Irradiation with X-ray/Protons in the Presence of PdPt NPs

**Figure 5:**
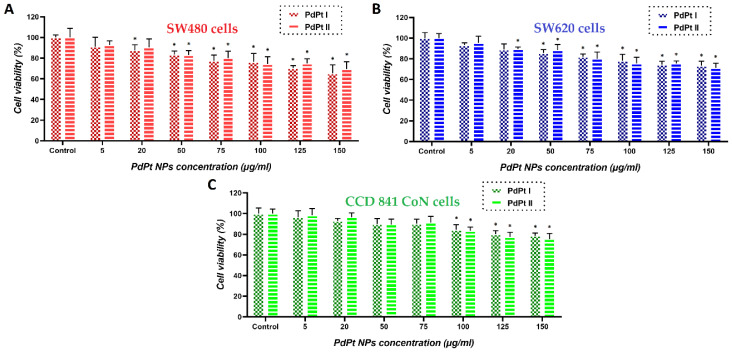
Cytotoxicity of PdPt (I) and PdPt (II) NPS against (**A**) SW480 (red bars), (**B**) SW620 (blue bars) and (**C**) CCD 841 CoN green bars) cells after 18 h of incubation. Differences were considered significant when * *p*-value < 0.05 vs. control.

The viability of cells treated with PdPt NPs, X-rays/protons and simultaneously with NPs and X-rays/protons was measured using the MTS test. The first step was to determine the viability of cancer (SW480 and SW620–from the primary tumor and metastatic lesion to lymph node, respectively) and normal (CCD 841 CoN) cells with respect to the concentration of PdPt NPs added. The results are shown in Figure 5. 

The above results indicate that for all analyzed cell lines, there was a tendency for a decreased cell viability in response to the increased concentrations of PdPt (I) and PdPt (II) NPs used, although at low concentrations of both PdPt NPs types, no statistically significant cytotoxic effect was noticed. For each cancer cell line, the maximum non-toxic concentration of PdPt NPs was selected individually and used to check their radiosensitizing potential: 50 µg/mL for SW480 cells and 75 µg/mL for SW620 cells. SW480 cells are generally less aggressive, less metastasizing and less sensitive to the NPs compared to SW620 cells [35,36]. Obviously, both cancer cell lines used were more sensitive to the treatment with NPs, than normal ones. There was no significant difference in cytotoxicity between nano-alloy and core-shell PdPt NPs used. Similar tests were performed for other incubation times of cells with NPs; however, there was no difference in cell viability after 3, 18, 24 and even 42 h of incubation times (data shown in the Supplementary Materials, Figure S1).

**Figure 6:**
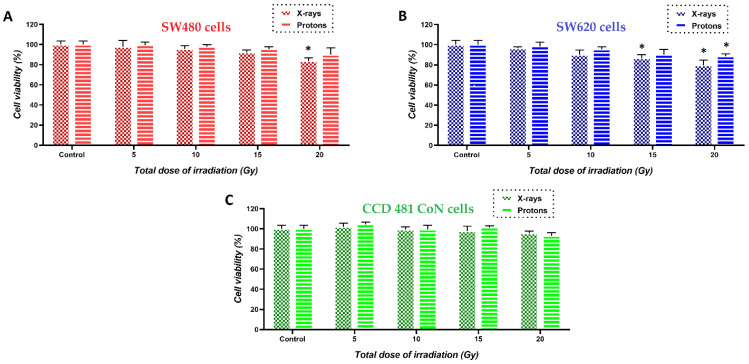
Viability of (**A**) SW480 (red bars), (**B**) SW620 (blue bars) and (**C**) CCD 841 CoN (green bars) cells treated with a different dose of X-rays and protons determined by the MTS test. Differences were considered significant when * *p*-value < 0.05 vs. control.

The next step was to determine the maximum non-harmful total doses of the photon (X-rays) and proton irradiation. Figure 6 summarizes the results. Higher doses of X-ray/proton radiation (15 and 20 Gy) caused slight damage to cancer cells, while normal cells remained viable after irradiation, even with a dose of 20 Gy. However, in further experiments, to assess the effect of the same treatment, the cells (cancer and normal) were irradiated with a dose of 15 Gy in order to have a comparison of whether this combined therapy would have the same effect on normal cells and cancer cells. 

**Figure 7:**
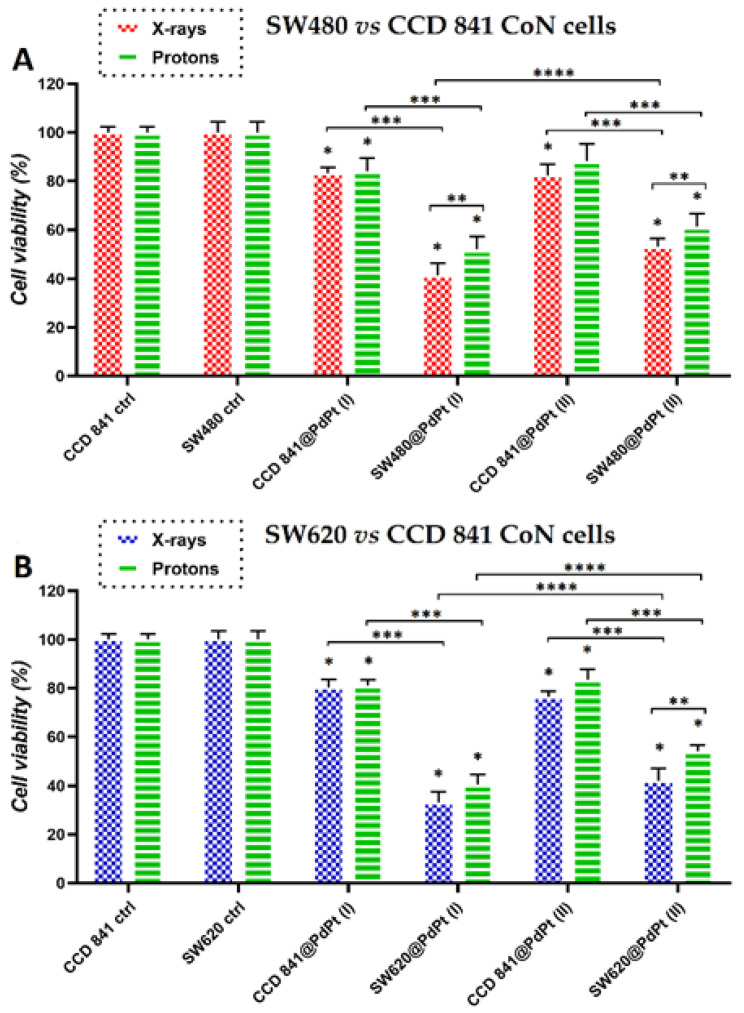
Viability of (**A**) SW480 and (**B**) SW620 compared to the viability of CCD 841 CoN normal colon epithelium cells after their incubation with PdPt (I) or PdPt (II) and X-ray (blue bars) or proton (green bars) irradiation with the total dose of 15 Gy. The MTS test was performed after 18 h of cell irradiation, preceded by an 18 h culture of cells with non-toxic concentrations of NPs. Data were considered significant if * *p*-value < 0.05 vs. control, ** *p*-value < 0.05—statistically significant differences between PdPt NPs-assisted X-ray and proton irradiation. *** *p*-value < 0.05—statistically significant differences between respective cancer and normal cells, **** *p*-value < 0.05—statistically significant differences between the radiosensitizing effect of PdPt (I) and PdPt (II).

Finally, the effectiveness of PdPt NPs in simulated X-ray or proton irradiation against cancer cells was investigated. Figure 7 summarizes the results of cell viability determined by the MTS test for all tested cell lines subjected to the combined treatment. 

The first conclusion that we can draw is the fact that PdPt NPs-assisted photon/proton irradiation has larger therapeutic efficacy compared to conventional irradiation. Importantly, from the therapeutic point of view, CCD 841 CoN cells were only slightly damaged; their survival after PdPt NPs-assisted irradiation (for both types of NPs and types of irradiation) was 82–86%. In general, bimetallic PdPt NPs proved to be better radiosensitizers in simulated X-ray than proton radiotherapy. However, this only applies to cancer cell lines. The survival of SW480 cells treated with X-rays^*^/protons^#^ was 41%^*^/52%^#^ and 53%^*^/61%^#^ for PdPt (I) and PdPt (II) NPs, respectively. In turn, for metastatic SW620 cells irradiated with X-rays^*^/protons^#^, the cell viabilities were 33%^*^/40%^#^ and 42%^*^/53%^#^ for PdPt (I) and PdPt (II) NPs, respectively. Although the radiation doses used for X-rays and protons were the same, they differed in energy. The high-energy protons (225 MeV) used in our experiment are characterized by a relatively small cross-section, which is the probability of a nuclear reaction–in this case, the reaction between protons and the high-Z metal PdPt NPs [37]. At lower radiation energies, the chance for such a reaction is much higher, and hence the physical mechanism of radiosensitization (by generating, e.g., gamma radiation or secondary electrons) could be more involved [38]. In turn, for X-rays, the photoelectric effect, as a result of its interaction with PdPt NPs, is the predominant process. This interaction can result in the generation of secondary electrons, Auger electrons or the characteristic X-rays of platinum/palladium atoms [39]. The type of cell death may also be influenced by the radiation used–X-rays or protons. It has been shown in the literature that high-energy protons can cause necrosis more often than X-rays; in this case, apoptosis is the dominant mechanism of cell death [40]. 

**Figure 8:**
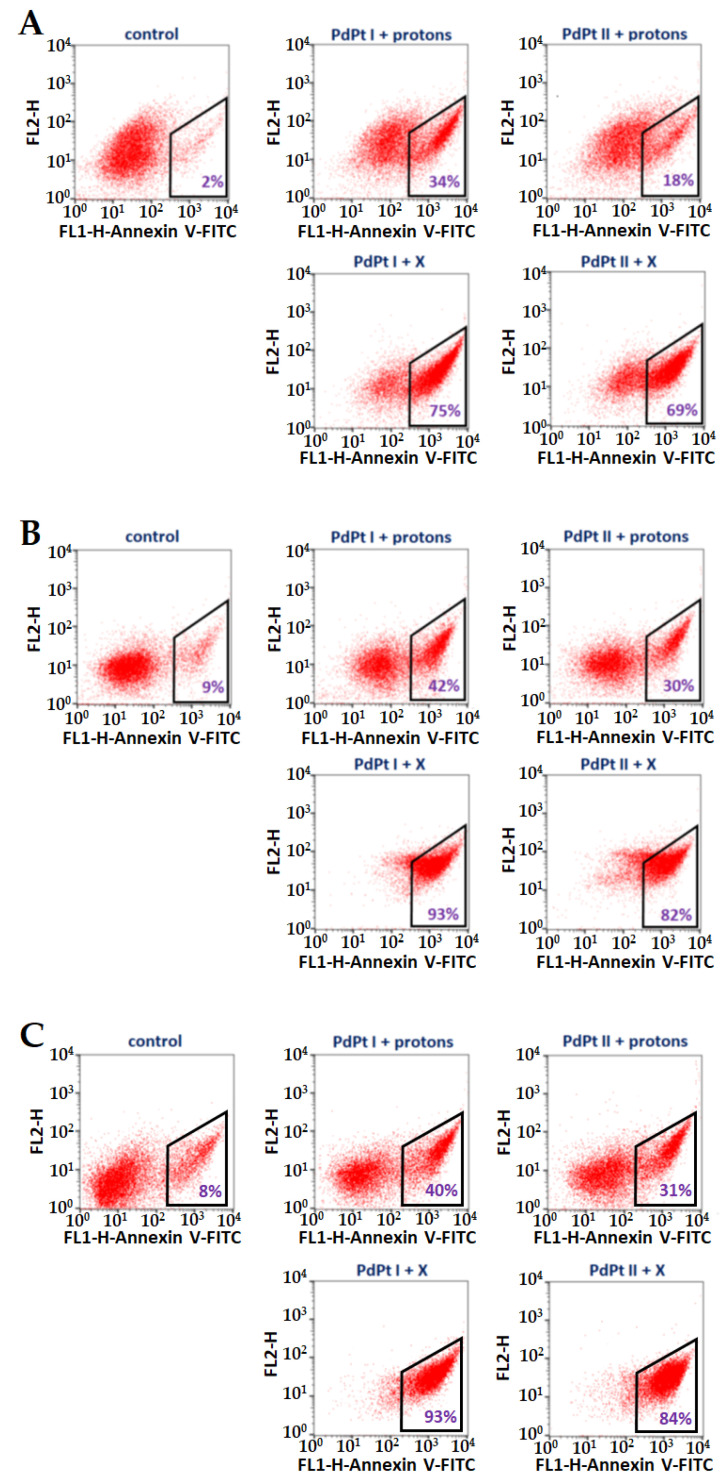
Flow cytometry analysis of Annexin V-binding - dot plots FL1-H vs. FL2-H show Annexin V-FITC stained (**A**) CCD 841 CoN, (**B**) SW480 and (**C**) SW620 cells after the addition of PdPt NPs, followed by proton or X-ray irradiation. The percentage of apoptotic cells is marked in violet.

The Annexin V-binding assay allowed to check the level of apoptotic cells after the addition of PdPt NPs and irradiation with X-rays/protons. The results (corresponding dot-plot) are shown in Figure 8A–C. These dot-plots show living (Annexin V-negative) and apoptotic (Annexin V-positive) cells. In general, the results of these studies are in line with cell viability data obtained using the MTS test, as a higher percentage of apoptotic cells of cancer origin was observed. This effect was less pronounced when protons were used; here, only 18% or 34% (depending on the used PdPt NPs) of apoptotic cells were detected for normal cells, in turn, for cancer cells, 30–42%. However, in the case of X-rays, significantly greater cell death, including normal cells, has already been observed. Such remarkable differences in the death rate of cells undergoing various types of radiation may be due to, for example, the fact that some of the cells irradiated with protons could undergo necrosis and not apoptosis. 

The very promising radiosensitizing properties of both PdPt NPs are related to their morphology. As shown in the STEM images (Figure 3A1,B1), both PdPt NPs have a rough, strongly developed surface. Such a high surface-to-volume ratio ensures better contact of PdPt NPs with the target cells. Moreover, the radiation interacting with NPs generates an increased amount of ROS, which is responsible for the biological-chemical mechanisms of radiosensitization [41,42,43]. Comparing both types of bimetallic PdPt NPs obtained in the frame of this work, it must be stated that approximately 10% more cancer cells die when PdPt (I) NPs are used as radiosensitizers (in both simulated photon and proton radiotherapy). For these two types of PdPt NPs, the shape, size, synthesis method and value of the zeta potential were similar, so the reason for the difference in their radiosensitizing properties can be the result of the distribution of platinum and palladium atoms. In the case of randomly distributed platinum and palladium atoms in nano-alloys PdPt NPs, the cells were in contact with both types of metals, which presumably determined the combined effect of such a treatment. Alternatively, the combined effect of platinum and palladium may not be present, and the more promising properties of PdPt I may only be due to the presence of palladium atoms on the surface of these NPs, which may possess better radiosensitizing properties than platinum. The differences in the estimated diameters of both types of NPs are of the order of several nm; such slight differences certainly do not affect (and if so–to a minimal extent) their cytotoxicity and photo- or radiosensitizing properties [44,45,46]. 

The important role of gallic acid in the synthesis of these NPs should also be emphasized. Although we have no formal proof, it cannot be ruled out that such a high selectivity of this type of in vitro treatment of cancer cells was also related to gallic acid as a PdPt NPs stabilizer; the strong anticancer properties of this compound have been already documented [47,48]. It cannot be forgotten that the naked, non-functionalized noble metal NPs used in this study are characterized by poor biodegradability. After NPs delivery to the body, they accumulate in the reticuloendothelial system (particularly in the liver and spleen). Upon internalization of such NPs into cells, they can be partially digested by lysosomal enzymes, resulting in the release of relatively toxic metal ions. These ions can influence cell homeostasis, giving an enhanced cytotoxic effect. Therefore, prior to in vivo studies, these NPs ought to be functionalized appropriately to give them more desirable pharmacokinetic and pharmacodynamic properties [49,50]. Exemplary, modifying NPs with different polymers can increase their time in blood circulation (dextran), reduce NPs toxicity (polylactide acid, alginate) or increase the hydrophilicity of NPs (polyethylene glycol) [51]. Unfortunately, due to the fact that NPs in the body can release toxic ions, which inactivate important vital enzymes, it is necessary to coat these NPs with biocompatible substances, e.g., silica or galactose [52,53]. 

## 4. Conclusions

In this work, we analyzed the influence of the structure of bimetallic PdPt NPs on their potential application with X-rays or proton beams as radiosensitizers in cancer cell irradiation. In vitro studies have shown that this type of simulated, combinational treatment selectively eliminates cancer cells, inducing a low death rate of normal cells. It turned out that nano-alloy bimetallic NPs are better radiosensitizers than their PdPt core-shell counterparts. The final effect of this type of PdPt NPs-supported irradiation was particularly strong when using X-rays–about 10% more cancer cells died when cell cultures were irradiated in the presence of NPs with X-rays compared to the proton beam. Importantly, from the ecological point of view, it is essential to use green reagents for the preparation of these nanomaterials. We expect that continued research on the use of nano-radiosensitizers to improve cancer therapy will result in finding a golden means to fight this disease. Indeed, nanotechnology gives us much wider possibilities to tackle cancers, such as targeted anticancer drug delivery, e.g., using smart thermo-, pH- or redox-sensitive drug delivery systems that release the drug directly in the vicinity of the tumor. 

## Figures and Tables

**Table 1:** Zeta potential values depending on pH for both types PdPt NPs.

pH	Zeta Potential–PdPt (I) (mV)	Zeta Potential–PdPt (II) (mV)
3	−8 ± 1	−8 ± 1
5	−15 ± 2	−8 ± 3
7	−16 ± 2	−9 ± 2
9	−10 ± 2	−8 ± 2
11	−11 ± 2	−11 ± 3

## Data Availability

Not applicable.

## Supplementary Materials

The following supporting information can be downloaded at: https://www.mdpi.com/article/10.3390/cancers14235899/s1, Figure S1: Exemplary results of the MTS viability test for the SW480 cell line cultured with both types of PdPt NPs for (A) 3 h, (B) 24 h and (C) 42 h.
