# Supplementary material for: Improving the Effect of Cancer Cells Irradiation with X-rays and High-Energy Protons Using Bimetallic Palladium-Platinum Nanoparticles with Various Nanostructures
Source: Cancers (Basel). 2022 Nov 29;14(23):5899. doi: 10.3390/cancers14235899 (PMC9736524; doi:10.3390/cancers14235899)
Supplement: Supplementary file 1 [file cancers-14-05899-s001.zip › cancers-2071112-supplementary.pdf]

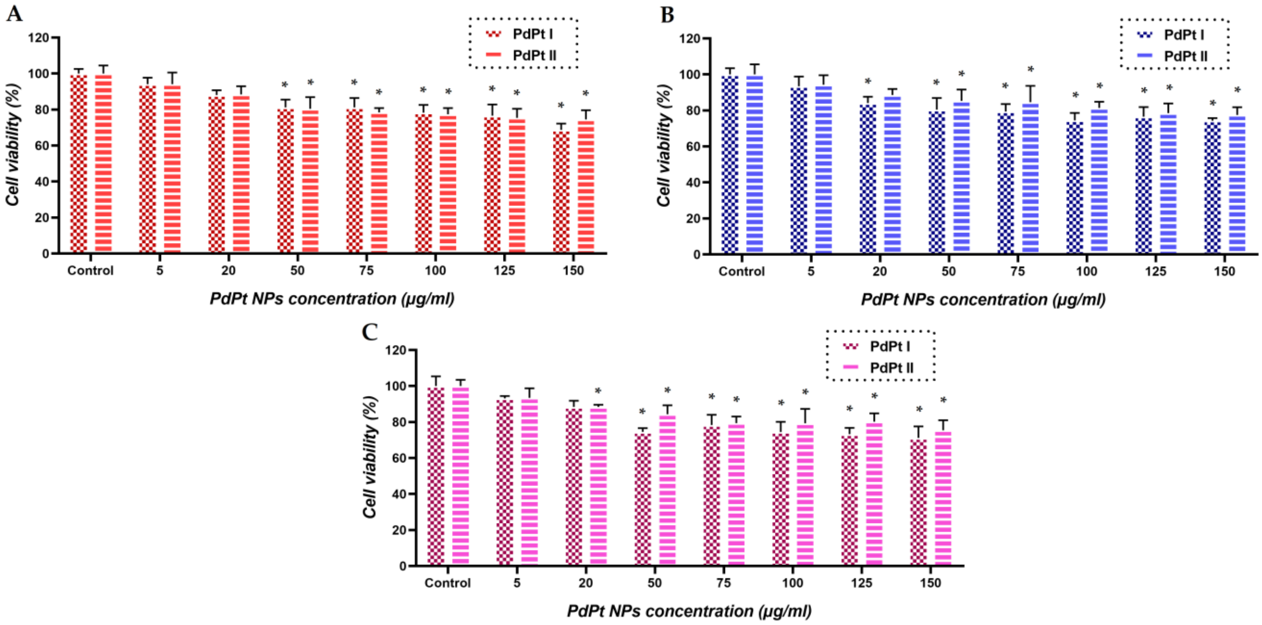

Figure S1. Exemplary results of the MTS viability test for the SW480 cell line cultured with both types of PdPt NPs for (**A**) 3 h, (**B**) 24 h and (**C**) 42 h.
